# Schisandrin B reverses doxorubicin resistance through inhibiting P-glycoprotein and promoting proteasome-mediated degradation of survivin

**DOI:** 10.1038/s41598-017-08817-x

**Published:** 2017-08-21

**Authors:** Shengpeng Wang, Anqi Wang, Min Shao, Ligen Lin, Peng Li, Yitao Wang

**Affiliations:** 1 State Key Laboratory of Quality Research in Chinese Medicine, Institute of Chinese Medical Sciences, University of Macau, Macao, 999078 China; 2 Department of Bioengineering, Zunyi Medical University Zhuhai Campus, Zhuhai Guangdong, 519041 China

## Abstract

Acquired drug resistance poses a great challenge in cancer therapy. Drug efflux and anti-apoptotic processes are the most two common mechanisms that confer cancer drug resistance. In this study, we found that Schisandrin B (Sch B), one of the major dibenzocyclooctadiene derivatives extracted from Chinese herbal medicine Schisandrae Chinensis Fructus, could significantly enhance the sensitivity of doxorubicin (DOX)-resistant breast cancer and ovarian cancer cells to DOX. Our results showed that Sch B increased the intracellular accumulation of DOX through inhibiting expression and activity of P-glycoprotein (P-gp). Meanwhile, Sch B could markedly downregulate the expression of anti-apoptotic protein survivin. Overexpression of survivin attenuated the sensitizing effects of Sch B, while silencing of survivin enhanced Sch B-mediated sensitizing effects. Furthermore, Sch B preferentially promoted chymotryptic activity of the proteasome in a concentration-dependent manner, and the proteasome inhibitor MG-132 prevented Sch B-induced survivin downregulation. Taken together, our findings suggest that Sch B could be a potential candidate for combating drug resistant cancer via modulating two key factors that responsible for cancer resistance.

## Introduction

Drug resistance is a major obstacle to most chemotherapeutic agents in clinical use, rendering tumor relapse and chemotherapy failure. Increased cellular drug efflux is one of the most recognized mechanisms of resistance that cause reduced drug concentrations in cancer cells^1^. Much effort has focused on the development inhibitors of ATP-binding cassette (ABC) drug transporters, mainly P-glycoprotein (P-gp), multidrug resistance associated proteins (MRPs) and breast cancer resistance protein (BCRP), however, clinical trials studying this paradigm have mostly failed^2, 3^.

In many cancers, the imbalance between pro- and anti-apoptotic processes also leads to apoptosis resistance against different chemotherapeutic agents^4^. Cancer cells are often found to overexpress many anti-apoptotic molecules that enable cells to escape programmed cell death^5^. The inhibitors of apoptosis (IAPs), including XIAP, ILP2, NAIP, livin, BRUCE, c-IAP1, c-IAP2 and survivin^6^, are found to be overexpressed in several resistant cancers and confer protection against apoptosis. As the smallest member of the IAP family, survivin (16.5 kDa) is widely expressed in various human cancers while undetectable in most normal adult tissues^7, 8^. Survivin is a nodal protein that interferes with the procedure of cell apoptosis regardless of which pathway apoptosis is originated^9^. As an attractive target for cancer therapy, survivin-targeted therapies, or in combination with other therapeutic approaches, still deserve further investigation in cancer resistance therapy^10^.

**Figure 1:**
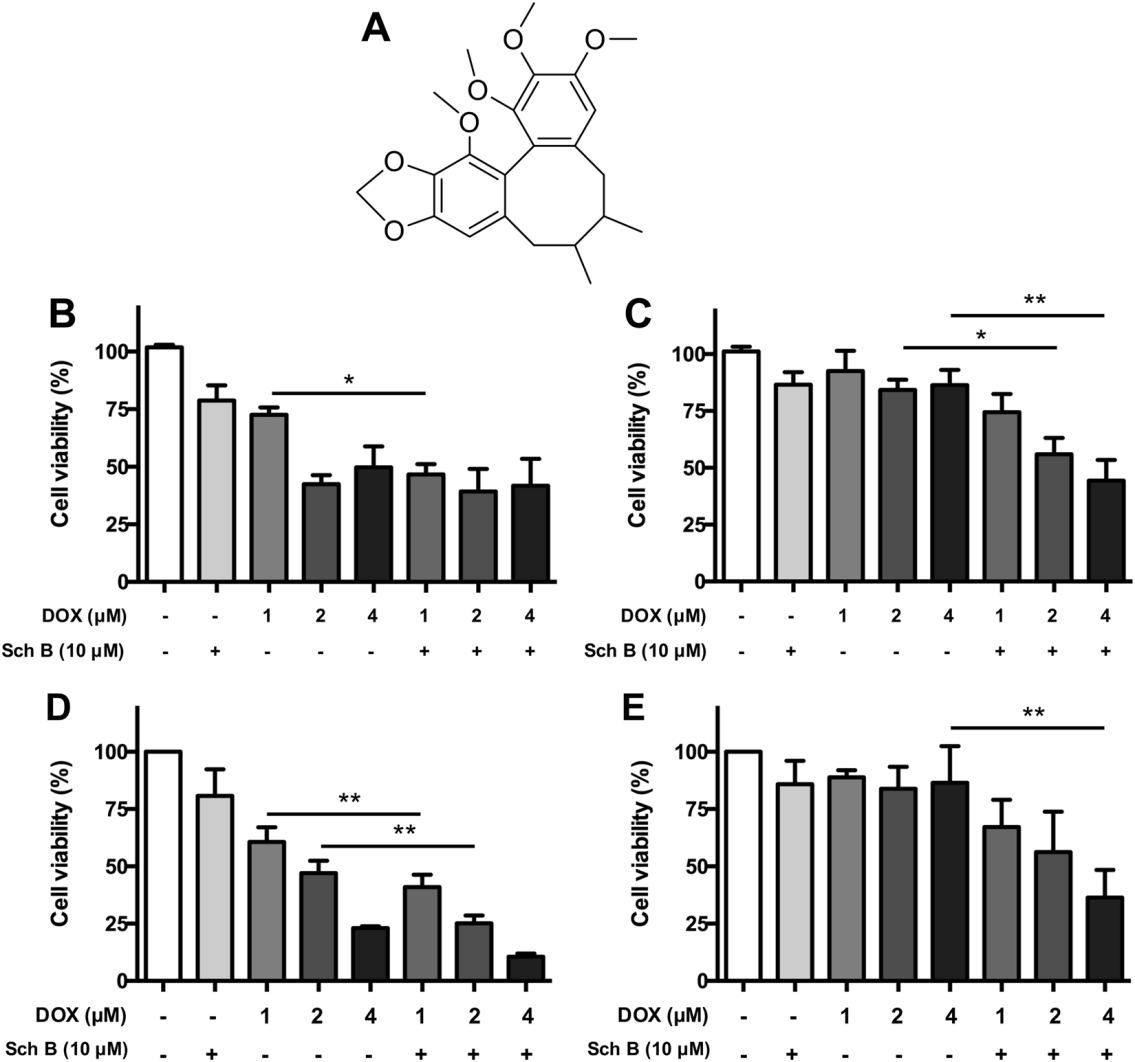
Sch B synergistically enhances cytotoxicity of DOX in DOX-resistant cancer cells. (**A**) Chemical structure of Sch B. MCF-7 (**B**), MCF-7/ADR (**C**), A2780 (**D**) and A2780/DOX (**E**) cells were treated with DOX for 48 h with or without the pretreatment of Sch B (10 μM) for 12 h and the cell viability was determined by MTT assay. All values represent mean ± SEM. *P < 0.05 and **P < 0.01.

Schisandrin B (Sch B, Fig. 1A), the major bioactive component isolated from Chinese medicine Fructus Schizandrae, is a dibenzocyclooctadiene lignan that possesses multiple biological activities. It is reported that Sch B could protect doxorubicin (DOX)-induced cardiac dysfunction^11^, attenuate acetaminophen-induced hepatic injury^12^, and also attenuate tertbutylhydroperoxide-induced cerebral toxicity^13^. Meanwhile, Sch B was identified as a potent P-gp inhibitor and could enhance DOX-induced apoptosis in cancer cells, but not in primary rat cardiomyocytes and primary human fibroblasts^14, 15^. Sch B can also inhibit MRP1, even stronger than the MRP1 inhibitor probenecid at the equimolar concentration^16^. Furthermore, Sch B can suppress cancer metastasis^17^, induce cell cycle arrest^18^ and inhibit ATR protein kinase activity^19^. These abundant evidences suggest that Sch B may play both protective and sensitizing roles in cancer treatment, but its potential and underlying mechanism for reversing cancer resistance still require further investigation. In the present study, we aimed to investigate the potential of Sch B in reversing cancer drug resistance and explore its underlying mechanisms of action.

## Results

### Sch B synergistically enhances cytotoxicity of DOX in DOX-resistant cancer cells

Firstly, the combination effect of Sch B and DOX was evaluated by MTT assay. Treatment with DOX significantly decreased the viability of MCF-7 cells, while DOX did not affect the viability of DOX-resistant MCF-7/ADR cells. The drug resistance index (DRI) of DOX-resistant MCF-7/ADR cells was calculated to be 25.59. Treatment with 2 μM of DOX evoked an inhibition of more than 50% cell viability in MCF-7 cells (Fig. 1B), while only about 10% decrease of cell viability was observed in MCF-7/ADR cells (Fig. 1C), indicating that the MCF-7/ADR cell line was resistant to DOX. Treatment with Sch B (10 μM) for 12 h prior to DOX treatment obviously enhanced the cytotoxicity in MCF-7/ADR cells. As shown in Fig. 1B and C, 10 μM of Sch B induced 18.6% and 13.4% decrease of viability in MCF-7 and MCF-7/ADR cells, respectively. When combined Sch B with various concentrations of DOX, the viability of MCF-7/ADR cells was decreased in a concentration-dependent manner, which was not observed in MCF-7 cells. To investigate the interactions between the DOX and Sch B, the combination index (CI) was further calculated. The respective CI values of Sch B combining with 1, 2 and 4 μM DOX were 0.486, 0.200 and 0.127 in MCF-7/ADR cells, indicating that Sch B synergistically enhanced cytotoxicity of DOX in DOX-resistant MCF-7/ADR cells.

To further confirm the effect of Sch B in reversing DOX resistance, we then evaluate the combination effect of Sch B and DOX in DOX-resistant ovarian cancer cells. DOX significantly decreased the viability of A2780 cells in a concentration-dependent manner (Fig. 1D), while treatment with 4 μM of DOX for 48 h barely decreased the cell viability of A2780/DOX cells to 86.4% (Fig. 1E), indicating that the A2780/DOX cell line was resistant to DOX. However, pretreatment with Sch B (10 μM) for 12 h significantly enhanced the cytotoxicity of DOX in A2780/DOX cells. Combination of Sch B with 1, 2 and 4 μM DOX decreased the viability of A2780/DOX cells to 67.2%, 56.2% and 36.4%, respectively. The CI values of Sch B combining with 1, 2 and 4 μM DOX were 0.219, 0.118 and 0.041 in A2780/DOX cells, showing that the combination of Sch B and DOX was synergistic against DOX-resistant ovarian cancer cells.

### Sch B enhances DOX-induced apoptosis in DOX-resistant cancer cells

**Figure 2:**
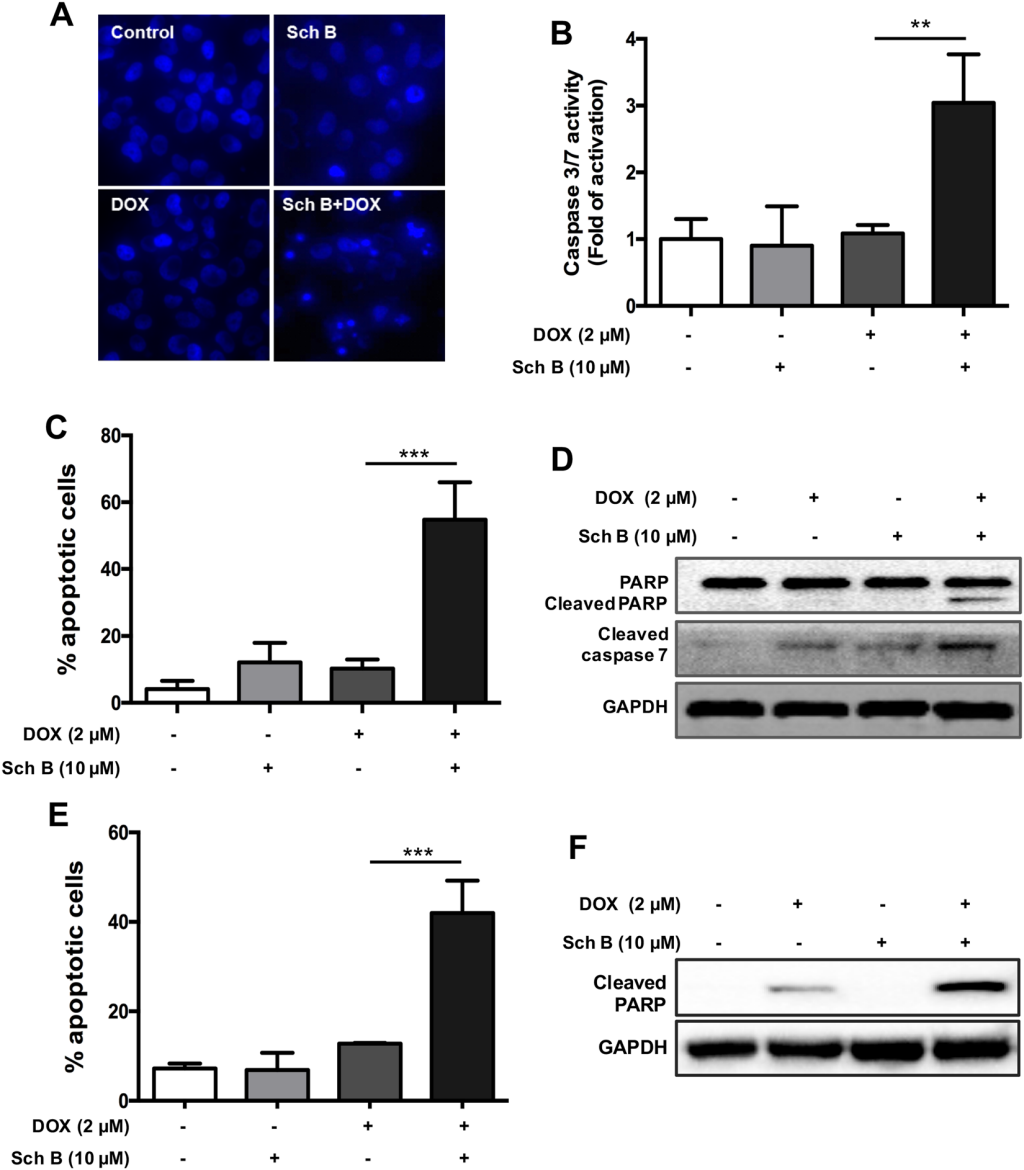
Sch B enhances DOX-induced apoptosis in DOX-resistant cancer cells. (**A**) MCF-7/ADR cells were treated with DOX for 48 h with or without the pretreatment of Sch B (10 μM) for 12 h. Cells were fixed and stained with Hoechst 33342 (1 μg/mL) and nucleus morphology analysis was performed using Incell Analyzer 2000 (GE Healthcare). (**B**) Caspase-3/7 activity of MCF-7/ADR cells was tested as described above. MCF-7/ADR (**C**) and A2780/DOX (**E**) cells were treated as described above and stained with Annexin V/PI and then cell apoptosis was analyzed by flow cytometry. **P < 0.01 and ***P < 0.001. Western blotting of lysates from MCF-7/ADR (**D**) and A2780/DOX (**F**) cells that had been treated as described above using the indicated antibodies. Similar results were obtained in three separate experiments.

We further evaluated whether Sch B sensitized MCF-7/ADR cells to DOX through induction of apoptosis. As shown in Fig. 2, treatment with DOX did not obviously induce cell apoptosis in MCF-7/ADR cells, which was in keeping with the results of MTT assay. However, when treated with Sch B prior to DOX, the apoptotic rate was significantly increased. Apoptotic nuclei were clearly observed in the combination group but not in other groups (Fig. 2A). It was also observed that combination treatment of DOX and Sch B led to a 3.04-fold of increase in activity of caspase-3/7 as compared with untreated control, while DOX and Sch B alone did not significantly increase caspase-3/7 activity (Fig. 2B). Annexin V-PI staining assay showed that combination of Sch B and DOX induced 54.8% of cell apoptosis, while DOX and Sch B induced much lower apoptotic rates (Fig. 2C). The results were further confirmed by observing the cleavage products of poly(ADP-ribose) polymerase (PARP) and caspase-7, which are markers of cell apoptosis^20^. Our results demonstrated that combination of DOX and Sch B stimulated the cleavage products of PARP and much higher level of lytic form of caspase-7 in MCF-7/ADR cells (Fig. 2D).

In DOX-resistant A2780/DOX cells, DOX treatment evoked cell apoptosis to 12.8%, whereas the combination of Sch B and DOX increased the apoptotic rate to 42.0%, 3.28-fold higher than that of DOX group (Fig. 2E). Similarly, Western blotting assay showed that DOX treatment resulted in low expression level of cleaved PARP, when combined with Sch B, the level of lytic form of PARP was further increased (Fig. 2F). Taken together, these results indicate that Sch B could enhance DOX-induced cell apoptosis in DOX-resistance cancer cells.

### Sch B increases the intracellular accumulation of DOX by inhibiting the expression and activity of P-gp

**Figure 3:**
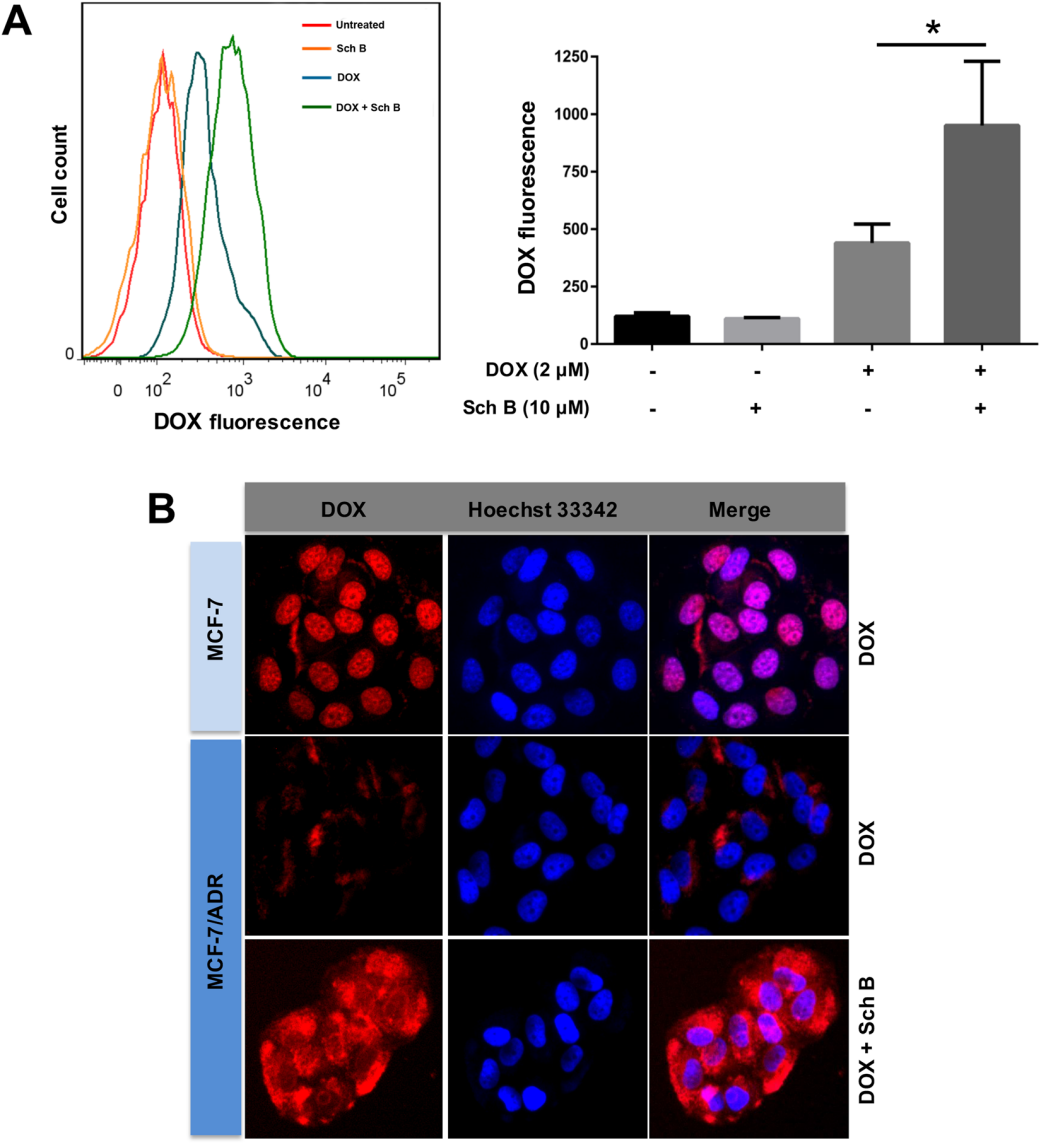
Sch B increases the intracellular accumulation of DOX. MCF-7 and MCF-7/ADR cells were pretreated with Sch B for 12 h and then incubated with 2 µM of DOX for 4 h. The intracellular level of DOX was determined by flow cytometry (**A**) and observed using Incell Analyzer 2000 (GE Healthcare) (**B**). *P < 0.05.

To better understand the potential mechanisms by which Sch B enhanced sensitivity of MCF-7/ADR cells to DOX, further research was performed on investigation of the intracellular accumulation of DOX. As shown in Fig. 3A, when cells were exposed to combination of DOX and Sch B, the intracellular levels of DOX were significantly higher compared to DOX alone (*P* < 0.05). The accumulation of DOX was also observed by fluorescence microscopy. The intracellular level of DOX in MCF-7/ADR cells was significantly lower than that in the MCF-7 cells. While when MCF-7/ADR cells were treated with DOX along with Sch B, the intracellular levels of DOX were significantly increased (Fig. 3B).

**Figure 4:**
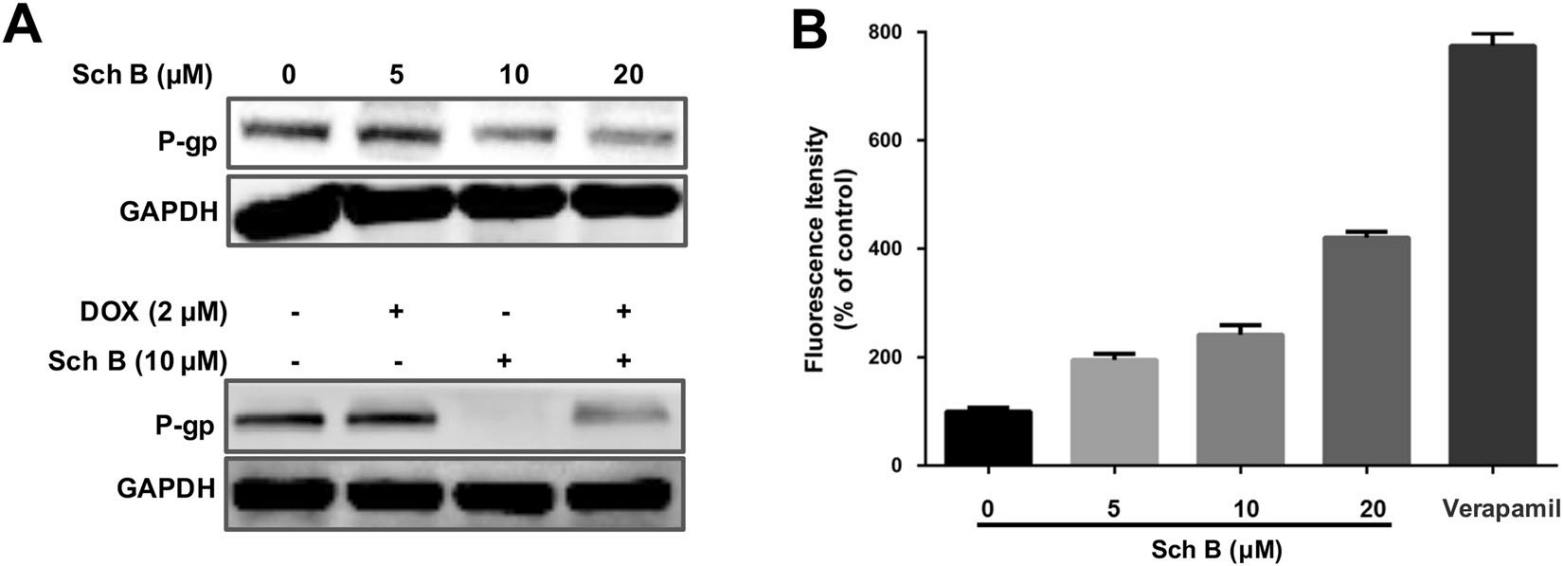
Sch B inhibits P-gp expression and activity. (**A**) Effects of Sch B on the expression of P-gp in MCF-7/ADR cells. After treatment with Sch B for 48 h, protein levels in cell lysates were analyzed by Western blot. Similar results were obtained in three separate experiments. (**B**) MCF-7/ADR cells was treated with different concentrations of Sch B for 12 h, then P-gp activity was determined using a fluorimetric MDR assay kit. The P-gp inhibitor verapamil (30 µM) was served as positive control. Results are expressed as mean ± SEM.

To confirm that P-gp was involved in the increased accumulation of intracellular DOX, the P-gp activity and expression level were further investigated. As shown in Fig. 4A, Sch B significantly inhibited the expression of P-gp in a concentration-dependent manner. Moreover, combination of DOX and Sch B also decreased the expression level of P-gp. We further measured the activity of P-gp using a fluorometric MDR assay kit, in which the P-gp inhibitor verapamil (30 µM) was served as positive control. Figure 4B showed that the intracellular intensity of fluorescent P-gp substrate in MCF-7/ADR cells was enhanced with the concentration of Sch B increased, meaning that Sch B inhibited P-gp activity in a concentration-dependent manner. Specifically, 5, 10 and 20 μM Sch B induced 1.95-, 2.42- and 4.20-fold increase of intracellular fluorescence intensity compared with the untreated control, respectively. Taken these results together, we deduced that Sch B played a great role in inhibition of the P-gp expression and activity and hence led to increased intracellular accumulation of DOX in MCF-7/ADR cells.

### Sch B inhibits survivin expression

**Figure 5:**
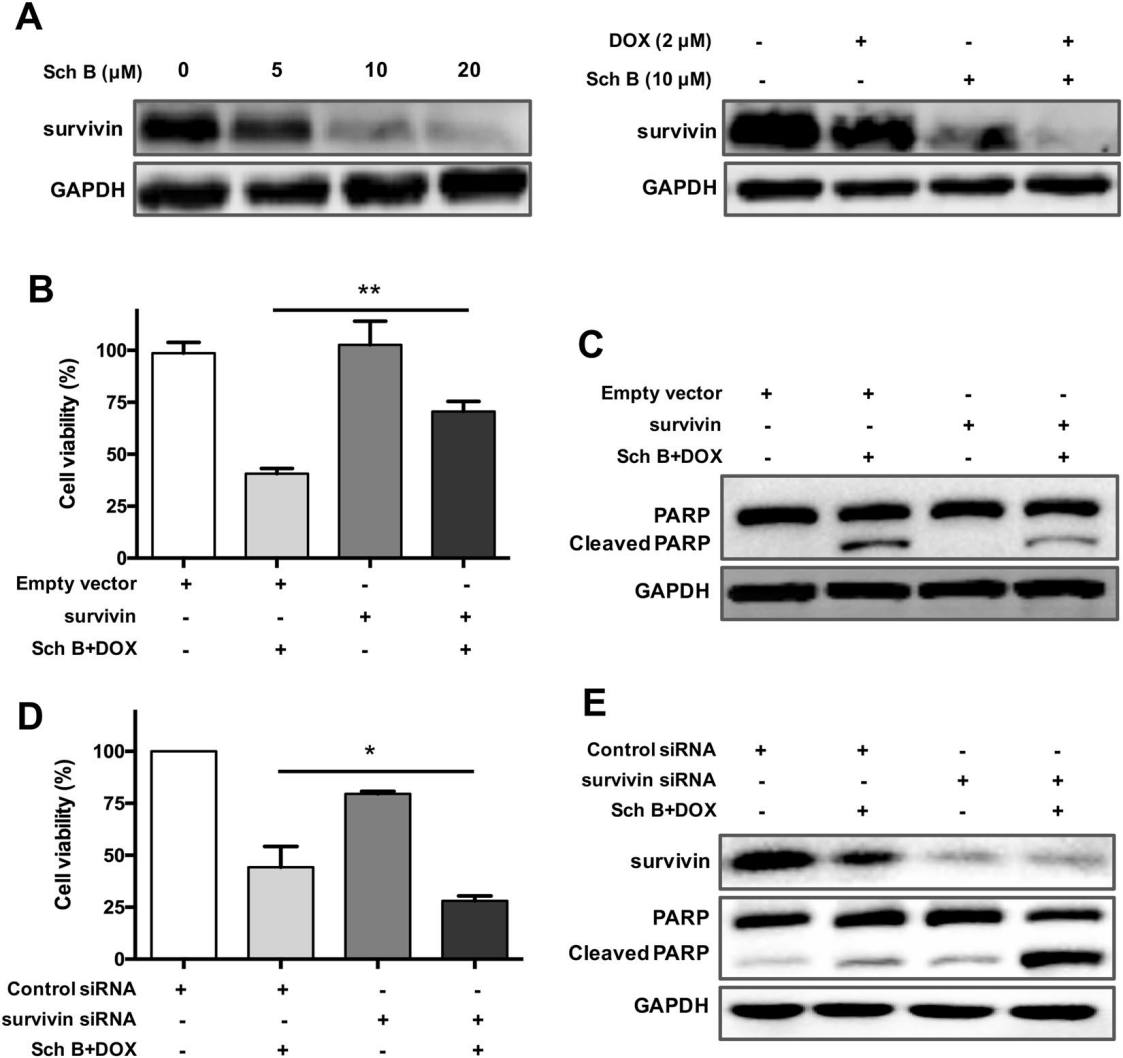
Inhibition of survivin is essential for Sch B-mediated sensitizing effect to DOX. (**A**) MCF-7/ADR cells were treated with different concentrations of Sch B (5, 10 and 20 μM) or in combination with DOX for 48 h. Survivin expression was evaluated using immunoblot assays. MCF-7/MDR cells were transfected with empty vector or plasmid encoding survivin, then cell viability was evaluated by MTT assay (**B**) and cleaved PARP level was analyzed by Western blot assay (**C**). MCF-7/MDR cells were transfected with control siRNA or survivin siRNA, then cells were subjected to MTT assay and Western blot assay to evaluate viability (**D**) and expression of survivin and cleaved PARP (**E**). Results are expressed as mean ± SEM. *P < 0.05 and **P < 0.01.

As Sch B augmented DOX-induced apoptosis in DOX-resistant cancer cells, and survivin family proteins are one of the molecular determinants of DOX resistance in organotypic human breast cancer^21^, we sought to investigate the effects of Sch B on the expression of survivin. As illustrated in Fig. 5A, the expression of survivin was decreased in a concentration-dependent manner after treatment with Sch B. Moreover, combination treatment of DOX and Sch B also significantly decreased the expression of survivin.

### Inhibition of survivin is essential for Sch B-mediated enhancement of apoptosis

We further took an investigation on whether Sch B facilitated DOX-induced apoptosis through inhibiting the expression of survivin. MCF-7/ADR cells were transiently transfected with empty vectors or plasmids containing wild-type survivin for 48 h as previously reported^22^. Cell viability assay showed that the cytotoxicity of combination treatment of DOX and Sch B in MCF-7/ADR cells was significantly attenuated in survivin overexpressed cells (Fig. 5B). Further, the cleavage product of PARP was dramatically reduced by overexpression of survivin in MCF-7/ADR cells (Fig. 5C). To further confirm whether survivin downregulation played a critical role in Sch B-mediated enhancement of apoptosis, the effects of specific siRNA was examined. Introduction of survivin siRNA further enhanced the cytotoxicity of DOX/Sch B combination (Fig. 5D) and increased the cleavage of PARP (Fig. 5E). These data suggested that inhibition of survivin might be one of the key mechanisms that regulating DOX/Sch B-induced apoptosis.

### Sch B promotes proteasome activity in DOX-resistant breast cancer cells

**Figure 6:**
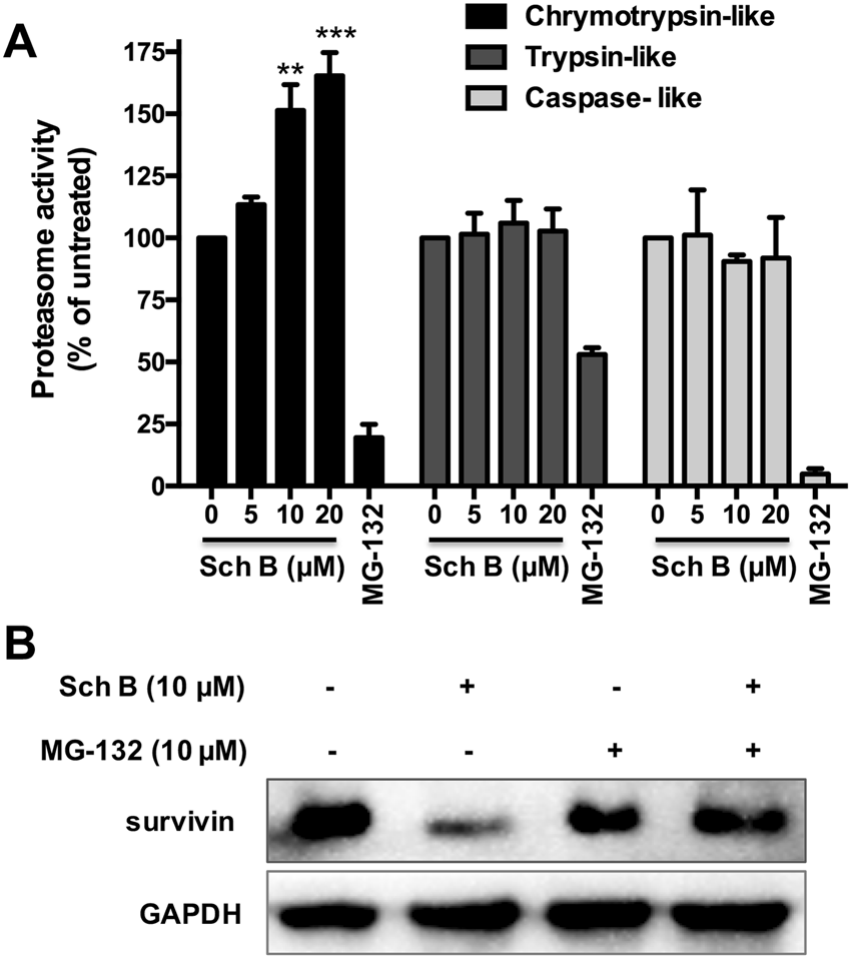
Sch B downregulates survivin through promoting proteasome activities in DOX-resistant breast cancer cells. (**A**) MCF-7/ADR cells were treated with the indicated concentrations of Sch B for 6 h and the proteasomal enzyme activities were analyzed using specific bioluminogenic enzyme substrates for chymotryptic (Suc-LLVY- aminoluciferin), tryptic (Z-LRR-aminoluciferin), and caspase-like activity (Z-nLPnLD-aminoluciferin). (**B**) Sch B-mediated downregulation of survivin was prevented by the proteasome inhibitor MG-132. MCF-7/ADR cells were preincubated with MG-132 (10 μM) for 1 h and then treated or not with 10 μM Sch B for further 24 h and survivin expression was assessed by Western blotting. One of three similar obtained results is shown. Results are expressed as mean ± SEM. **P < 0.01 and ***P < 0.001 as compared to that of the untreated control.

We then investigated whether regulation of proteasome activity was involved in Sch B-mediated downregulation of survivin. As shown in Fig. 6A, Sch B treatment preferentially promoted chymotryptic activity of the proteasome in a concentration-dependent manner in DOX-resistant MCF-7/ADR cells, while tryptic and caspase-like activities were not affected. Specifically, treatment of 5, 10 and 20 μM Sch B for 6 h increased the chymotryptic activity to 113.3%, 151.3% and 165.3%, respectively. In comparison to Sch B, the highly specific proteasome inhibitor MG-132 significantly decreased all three proteolytic activities of the proteasome. Moreover, the proteasome inhibitor, MG-132, prevented the Sch B-induced survivin downregulation (Fig. 6B).

## Discussion

Until now, many attempts have been made to restore the chemosensitivity of chemotherapeutic agents. In 1976, Rudy Juliano and his team workers discovered a membrane glycoprotein that could alter membrane permeability against chemotherapeutics, which became known as P-gp afterward^23^. Over the past 40 years, P-gp has been one of the most extensively studied membrane transporters that interact with over 300 compounds^24–26^. A large number of P-gp inhibitors have been discovered to inhibit the function of P-gp, however, unwanted immunosuppressive effects and unfavorable changes in the pharmacokinetics block their way into further clinical use^27, 28^. For these reasons, novel P-gp inhibitors, especially naturally occurring compounds, with the ability to interact with multiple targets have gained growing attention^29^. In this study, we identified Sch B as a potential agent for reversing DOX resistance through regulating both P-gp expression and activity. Moreover, Sch B is reported to protect DOX-induced side effects, including cardiac dysfunction^11^, indicating that Sch B may also exert protective effect when applied in combination with DOX.

Previous studies showed that patients whose tumors expressing survivin are correlated with poor survival, chemotherapy resistance, and increased tumor relapse^30^. Survivin is overexpressed almost 40-fold in tumors and renders cancer cells resistant to both chemotherapy and radiotherapy through inhibiting cell apoptotic function and stabilizing microtubule organization^7, 31, 32^. As one of the most cancer-specific targets, survivin plays a critical role in conferring cancer resistance and significant efforts have been focused on developing survivin-targeted therapies. However, clinical trials of survivin inhibitor YM155 and LY2181308 only achieved limited success, indicating that ample room remains for realizing the potential of targeting survivin to overcome cancer resistance^31, 33^. As a natural compound, Sch B was firstly identified in this study as a survivin inhibitor. Moreover, overexpression of survivin attenuated the effect of Sch B in reversing DOX resistance, thereby manifesting that suppression of survivin was a key mechanism through which Sch B sensitized DOX-resistant breast cancer cells to DOX.

It is previously reported that survivin is a short-lived protein and ubiquitin-proteasome pathway (UPP) is involved in the degradation of survivin^34, 35^. In eukaryotes, the UPP is the main non-lysosomal protein degradation pathway and plays key roles in the maintenance of cellular protein homeostasis^36^. As the heart of the UPP, 26 S proteasome endoproteolytically cleaves most intracellular proteins and thus regulates many crucial intracellular processes^37^. The 26 S proteasome is a large protein complex that contains a 20 S proteasome core particle for substrate proteolysis and two 19 S regulatory particles to recognize and unfold ubiquitin-tagged substrates^38^. Three β subunits of 20 S proteasome have hydrolytic activity and active sites are classified into chymotrypsin-like (CT-L), trypsin-like (T-L) and caspase-like (C-L) sites^39^. Previous studies demonstrated that selective inhibition of the chymotrypsin-like subunits of the proteasome could induce cell death in hematologically derived tumor cells^40^. ONX0912, a novel orally bioavailable chymotrypsin-like inhibitor, elicited an antitumor response equivalent to intravenously administered carfilzomib^41^. Targeting specific active sites of the proteasome is a prepossessing strategy for cancer therapy. Our results showed that Sch B preferentially promoted chymotryptic activities of the proteasome, and MG-132 prevented Sch B-mediated survivin downregulation, suggesting that inhibition of survivin was mediated by promoting survivin degradation in Sch B-treated MCF-7/ADR cells.

Resistance to chemotherapy still hamper the effective treatment ovarian cancer, causing the overall 5-year survival rate of only 10–30% for late-stage ovarian cancer^42^. Blocking drug transporters and lowering the threshold of apoptosis have shown to reverse ovarian cancer resistance^43, 44^. To verify the effect of Sch B in reversing DOX resistance, we further investigate this drug combination in DOX-resistant ovarian cancer cells in this study. We found that Sch B could also reverse DOX resistance in ovarian cancer cells though inhibiting P-gp and survivin. Our findings further support the development of Sch B for treatment of drug resistant cancers.

In conclusion, our data show that Sch B sensitizes DOX-resistant cancer cells to DOX-induced apoptosis. Sch B inhibits both the expression and activity of P-gp to increase the intracellular level of DOX. Meanwhile, Sch B preferentially promotes chymotryptic activity of the proteasome to facilitate UPP-mediated degradation of anti-apoptotic protein survivin. Our findings indicate that targeting P-gp and survivin may be a novel therapeutic strategy for breast and ovarian cancer that are resistant against DOX-based therapies. Further studies are, however, needed to define the pharmacokinetic properties of Sch B and the effects of the combined treatment with *in vivo* models of DOX-resistant cancer.

## Methods

### Materials

Sch B and DOX were purchased from Chengdu Must Bio-Technology Co. Ltd (Sichuan, China) and Meilun Biology Technology Co. Ltd (Dalian, China), respectively. 3-(4,5-Dimethylthiazol-2-yl)-2,5-diphenyltetrazolium bromide (MTT) and paraformaldehyde (PFA) were supplied by Sigma-Aldrich (St. Louis, MO, USA). Dulbecco’s Modified Eagle Medium (DMEM), fetal bovine serum (FBS), penicillin-streptomycin, 0.25% (w/v) trypsin/1 mM EDTA were purchased from Life Technologies (Grand Island, USA). The primary antibodies against PARP, cleaved PARP, cleaved caspase 7, survivin, P-gp and GAPDH were purchased from Cell Signalling Technology (Danvers, MA, USA). The proteasome inhibitor MG-132 and survivin siRNA were obtained from Santa Cruz Biotechnology (CA, USA). The water used was of ultrapure grade and was supplied by a Milli-Q purification system (Millipore Co., Billerica, MA, USA).

### Cell lines and cell culture

Human breast cancer MCF-7 cell line was obtained from the American Type Culture Collection (ATCC, Manassas, VA, USA). The A2780 human ovarian cancer and DOX-resistant A2780/DOX cell lines were obtained from European Collection of Cell Cultures (Salisbury, Wiltshire, UK). DOX-resistant MCF-7/ADR cells were selected in stepwise increasing concentrations of DOX as previously described^45, 46^. Both DOX-resistant MCF-7/ADR and A2780/DOX cells were incubated with 1 μM DOX every three passages to keep cells resistant to the drug. The cells were cultured in DMEM medium supplemented with 10% fetal bovine serum and penicillin/streptomycin (100 U/mL, 100 μg/mL) in a 37 °C, 5% CO_2_ incubator.

### Cell viability assay

Cell viability was assessed by MTT assay. Briefly, exponentially growing MCF-7, MCF-7/ADR, A2780, and A2780/DOX cells were seeded in 96-well plates and incubated overnight. After appropriate treatment, the cells were incubated with serum free medium containing MTT (1 mg/mL) for another 4 h. The formazan crystal was dissolved using DMSO and the spectrophotometric absorbance at 570 nm was determined by a microplate reader (SpectraMax M5, Molecular Devices, USA). The results were expressed as the percentage of viable cells over untreated control cells.

### Determination of intracellular DOX

The intracellular level of DOX was evaluated by flow cytometry and fluorescence microscopy. Briefly, MCF-7 and MCF-7/ADR cells were pretreated with Sch B for 12 h and then incubated with 2 µM of DOX for 4 h, and cells were carefully washed three times with PBS. For observation by fluorescence microscopy, cells were fixed with 4% PFA, washed again with PBS, stained with Hoechst 33342 (1 μg/mL), and imaged using Incell Analyzer 2000 (GE Healthcare Life Sciences, USA). Each condition was performed in triplicate. For flow cytometric analysis, cells were collected and washed trice with PBS, then analyzed using flow cytometer in FL2 PE channel. Generally, a total of 10,000 cells were collected, amplified, and scaled to generate single parameter histogram.

### Determination of P-gp activity

The activity of P-gp was determined by a fluorimetric MDR Assay kit (Abcam, Cambridge, UK). By following the user protocol provided by the vender, cells were seeded into 96-well flat clear bottom black-wall microplates and treated as indicated. The P-gp substrate verapamil was used as positive control. Then, 100 μL of MDR dye-loading solution was added to each well and the plate was incubated at room temperature for another 1 h in dark. Intracellular fluorescence was detected using a microplate reader at an excitation wavelength of 490 nm and emission of 525 nm. All experiments were performed in triplicate and compared to controls.

### Measurement of apoptosis

Apoptosis was evaluated by observing the nuclear morphology. After treatment, cells were washed twice with PBS and fixed with 4% PFA, followed by incubation with Hoechst 33342 (1 μg/mL) at room temperature for 15 min. Then observation of the apoptotic morphology of cell nuclei was performed on Incell Analyzer 2000 (GE Healthcare Life Sciences, USA). Apoptosis is characterized by nuclear condensation, cell shrinkage, membrane blebbing, and DNA fragmentation^47, 48^. Apoptosis rate was evaluated using an Annexin V-FITC Apoptosis Detection Kit (BioVision, Palo Alto, CA) by flow cytometry. Briefly, after appropriate treatment, the cells were collected by EDTA-free trypsin, washed twice with PBS and then stained with 5 μL of Annexin V-FITC and 10 μL of PI for 20 min at room temperature in the dark prior to flow cytometric analysis.

### Caspase activity assay

Caspase 3/7 activity were measured using the Caspase-Glo assay kit (Promega, Madison, USA). Cells were plated into 96-well white-walled plates and appropriately treated for 24 h. Subsequently, 100 μL of caspase assay reagent buffer was added to each well and incubated in the dark for 30 min. The bioluminescence was determined using a SpectraMax M5 microplate reader.

### Western blot

After appropriate treatment, cells were washed three times with ice-cold PBS and extracted by RIPA lysis buffer containing 1% PMSF and 1% Protease Inhibitor Cocktail for 30 min on ice, followed by centrifugation at 12,500 × g for 20 min at 4 °C. The protein concentration was determined using the BCA protein assay kit (Thermo Fisher Scientific, MA, USA). Equivalent amounts of protein samples were separated by SDS-PAGE, and then transferred to a polyvinylidene difluoride (PVDF) membrane (Bio-Rad, Hercules, CA, USA). After blocking with 5% (w/v) non-fat milk for 1 h at room temperature, the blots were incubated with primary antibodies overnight at 4 °C. After washing for three times, the membranes were further incubated with corresponding secondary antibodies for 1 h at room temperature. Finally, specific protein bands were visualized using ECL Plus Western blotting detection reagents (GE Healthcare, Piscataway, NJ, USA) and scanned by a ChemiDoc XRS Imaging system (Bio-Rad, CA, USA).

### Determination of proteasome activity

To determine the cellular proteasome activity, cells were seeded in white-walled 96-well cell culture plates, allowed to grow overnight, and then appropriately treated for 6 h. The proteasomal activity was analyzed using the Proteasome-Glo™ Cell-Based Assay system (Promega, Madison, USA) by adding 100 μL of Proteasome-Glo Lysis and reagent buffer containing Suc-LLVY-aminoluciferin, Z-LRR-aminoluciferin, or Z-nLPnLD-aminoluciferin for the chymotrypsin-like, trypsin-like and caspase-like activities, respectively. For trypsin-like assay, two additional inhibitors were added to the reagent buffer to reduce nonspecific protease activities. After 20 min of incubation at room temperature, the bioluminescence was detected using a SpectraMax M5 microplate reader.

### Statistical analysis

Statistical analysis was performed using the GraphPad Prism 6.0 statistical software (San Diego, CA, USA). The results were expressed as the mean of arbitrary values ± standard error mean (SEM). Statistical significance was assessed by one-way ANOVA followed by Tukey’s multiple comparison, where a *P*-value less than 0.05 denoted statistical significance. Interaction between the drug combinations was analyzed by the CI method according to the method of Chou and Talalay (1984)^49, 50^. CI less than 0.90 indicates synergism; CI between 0.90 and 1.10 indicates additivity; and CI more than 1.10 indicates antagonism. Data analysis was performed using Calcusyn software (Biosoft, Oxford, UK).
